# Synthesis and Characterization of Boehmite Particles Obtained from Recycling: Water Disinfection Application

**DOI:** 10.3390/nano12162771

**Published:** 2022-08-12

**Authors:** Dienifer F. L. Horsth, Julia de O. Primo, Nayara Balaba, Jamille S. Correa, Cristina M. Zanette, Douglas K. Silva, Carla Bittencourt, Fauze J. Anaissi

**Affiliations:** 1 Departamento de Química, Universidade Estadual do Centro-Oeste, Guarapuava 85040-167, Brazil; 2 Chimie des Interactions Plasma-Surface (ChIPS), Research Institute for Materials Science and Engineering, University of Mons, 7000 Mons, Belgium; 3 Departamento de Engenharia de Alimentos, Universidade Estadual do Centro-Oeste, Guarapuava 85040-080, Brazil

**Keywords:** aluminum, circular economy, green synthesis

## Abstract

We report on the synthesis of boehmite aluminum oxide hydroxide particles with lamellar structure (γ-AlO(OH)) obtained from the recycling of metallic can seals, with the addition of silver nanoparticles (Ag-NPs) reduced by *Aloe Vera* extract. X-ray diffractometry (XRD) confirmed the γ-phase, and scanning electron microscopy (SEM) showed the presence of Ag-NPs on the boehmite particle surface, confirming the efficiency of the synthesis to obtain the composite material. The samples were used to treat lake water, according to the Standard Methods for the Examination of Water and Wastewater. The results indicated that the elimination of total coliforms and *Escherichia coli* occurred, with excellent efficiency for the Ag-boehmite sample. The tests show the possibility of reuse (5×) of the sample, as it maintained the efficiency of disinfection for *E. coli*. The preparation, use, and reuse of boehmite obtained from metallic waste is a case of a circular economy, focused on sustainability and green chemistry.

## 1. Introduction

In the framework of environmental preservation, obtaining clean and potable water has become a challenge due to chemical and biological contaminations [1]. The access to drinking water is a right for all and is part of the sustainable development goals established by the United Nations [2,3]. However, around 40% of the global population still does not have access to clean water [4]. According to [3], untreated or inadequately treated water can cause diseases in humans due to the presence of microbial pathogens (bacteria, protozoan parasites, viruses, and others) [5]. The main pathogens found in water are bacteria (*E. coli*, *Shigella*, and *V. cholera*), viruses (Hepatitis A, Polio Virus, and Rota Virus), and parasites (*E. histolytica*, *Giardia*), responsible for diseases such as cholera, typhoid, paratyphoid, salmonella, cryptosporidiosis, giardiasis, etc. [6]. About 2.2 million annual deaths and threats to the lives of children worldwide are attributed to the ingestion of water contaminated by pathogens [7]. Therefore, the engineering of materials capable of disinfecting and sanitizing water is urgent [8]. The use of metal nanoparticles for disinfection and water sanitization have gained importance, and at the forefront are silver nanoparticles (Ag-NPs), due to their antimicrobial properties, potential for the elimination of heavy metals, and catalytic activity against azo dyes [9,10,11]. In its elemental form, silver (Ag) is toxic, though quite efficient in combating microorganisms. Nevertheless, the deposition of Ag-NPs on the surface of nanostructured oxides was reported to decrease their toxicity and to increase their bactericidal efficiency, as the surface area of nanoparticles dispersed on a nanostructured surface allows greater accessibility to microbes [9,10,12,13,14,15,16,17].

Considering the need for greener synthesis methods and the development of sustainable materials for the preservation of the environment, we report on the synthesis of Ag-NPs using Aloe Vera extract as a reduction agent, and their deposition on the surface of boehmite aluminum oxyhydroxide (γ-AlO(OH)) particles, obtained from aluminum recycling metal. The Ag-Boehmite nanoparticles were tested for lake water treatment. The lake water contamination arises from unauthorized water discharges from farms and houses in the neighborhood of the lake.

Research on materials has been focusing on green synthesis methods, aiming at the engineering of environmentally friendly materials [18,19,20]. The “greener synthesis” or “biosynthesis” of materials has become increasingly popular due to the current problems of toxicity and global pollution [21,22,23]. Green synthesis seeks to minimize waste, reduce pollution, use solvents and renewable resources, and to increase efficiency and environmental adaptability [24]. In this respect, the use of plant extracts such as *Aloe Vera*, in the synthesis of nanoparticles has been highlighted [25], because the plant extract can be used as a reducing agent and stabilizer of nanoparticles [16,26].

Aluminum is a nontoxic, resistant, malleable metal and, when oxidized, forms a protective layer on its surface. These properties make metallic aluminum ideal in several areas, such as the food, health, beauty, and automotive industries [27,28]. In this context, recycling aluminum cans has been of interest to companies around the world with direct investments to obtain sustainable and low-cost products [15]. In addition, recycling aluminum is a way to reduce the amount of byproducts linked to the production of primary aluminum discarded in the environment [27]. For every kilo of secondary (recycled) aluminum, about 5 kilos of bauxite are saved, and about 95% of the energy needed to produce the same amount of primary aluminum is saved [29]. The simplest recycling process consists of melting the metal [30]; however, chemical treatments can also be used to recycle aluminum [29]. The most used route to obtain aluminum oxide and hydroxide is the Bayer process, which explores the solubility of these compounds present in bauxite in a highly alkaline medium [31]. In this way, the possibility of transforming metallic aluminum into oxide or hydroxide is presented, exploring its solubility. The conditions of the precipitation of aluminum hydroxide (Al(OH)_3_) directly affect the hydroxide type formed, since it can exist in different crystalline forms, in addition to the amorphous phase, and this influences the characteristics of the product, and consequently its application. Crystalline hydroxides are divided into two classes, aluminum trihydroxides (Al(OH)_3_) and aluminum oxyhydroxides (AlO(OH)) [32]. A phase that stands out is boehmite (γ-AlO(OH)), as it has a lamellar structure, and can be obtained via a simple, low-cost method, using commercially available reagents, nontoxic in aqueous media, and without the need for an inert atmosphere [33]. Furthermore, due to its stable orthorhombic structure, it has properties such as insensitivity to variations in climatic conditions, high mechanical and thermal stability, high dispersity of active phases, nontoxicity, and easy surface modification [34,35].

## 2. Materials and Methods

### 2.1. Boehmite (γ-AlO(OH)) by Metallic Aluminum Acid Digestion

**Figure 1:**
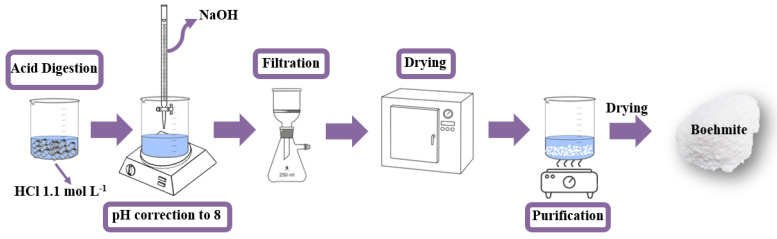
Schematic diagram of the synthesis route used to obtain the boehmite (γ-AlO(OH)).

Aluminum can seals (10.0 g) were digested in an acid solution (1.1 mol L^−1^, 1000 mL) of hydrochloric acid (HCl, P.A., Synth) for about 24 h (Figure 1). The final solution containing the Al^3+^ ions had a pH 0.5 [29]. To obtain the boehmite phase, the pH was corrected by adding sodium hydroxide (NaOH, Vetec, 3.0 mol L^−1^) until the pH reached 8, causing a white solid to precipitate. The solid was vacuum filtered and oven-dried at 70 °C for 24 h [29].

### 2.2. Sample Purification

During the process of obtaining the boehmite phase (Section 2.1), the formation of sodium chloride (NaCl) occurred, which was not of interest to the final application. Therefore, it was necessary to purify the boehmite powder. Purification was performed by exploring the solubility of NaCl, which is different from boehmite. Thus, the boehmite powder was washed with hot water (90 °C) for the solubilization and removal of sodium chloride. After this step, the powder was filtered and dried at 70 °C [29].

### 2.3. Ag-NPs’ Preparation with Aloe Vera Extract

To obtain the Aloe Vera extract, the plant leaves were washed with water and cut into small pieces, and used as a whole (peel and gel). Then they were boiled in deionized water, in the proportion of 5.0 g of Aloe Vera for each 100 mL of water. After the first boiling, the cubes were compressed with the aid of a pestle and boiled again for an additional 5 min. The extract was filtered and stored under refrigeration (5 °C). Ag-NPs’ formation was obtained by the addition of silver nitrate (Ag(NO_3_)·6H_2_O, P.A.-ACS, Synth) to the Aloe Vera extract (50 mL). The extract/silver solution remained in mechanical agitation (500 rpm) for 20 h, and after this period, the solution showed a brown/black color, indicating that it had formed Ag-NPs [26].

### 2.4. Boehmite Decorated with Ag-NPs

**Figure 2:**
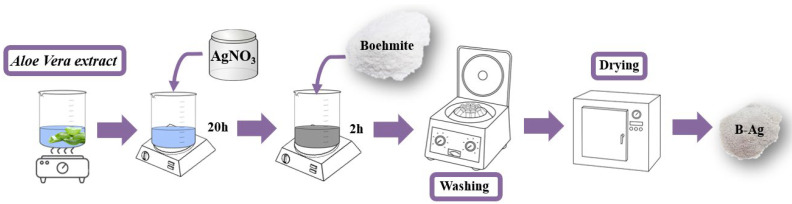
Schematic diagram of the synthesis route used to obtain Ag-boehmite (Ag-(γ-AlO(OH))).

The decoration of the boehmite surface was carried out by adding it to the solution containing the Ag-NPs and was agitated for 2 h (Figure 2). Then, the solution was filtered and washed five times with deionized water and oven-dried at 70 °C.

### 2.5. Characterization

The samples were characterized by X-ray diffraction (XRD), carried out in a Bruker D2 Phaser Diffractometer (Berlin, Germany), which uses a copper cathode with copper kα emission (λ = 1.5418 Å), equipped with a LynxEye high-performance detector, 300 W power. Scanning electron microscopy (SEM) images were obtained using a scanning electron microscope, SEM-TM3000, coupled with SwiftED 3000 (Energy Dispersive X-ray Spectrometer) and a Su8020 both from HITACH (Tokyo, Japan). Visible spectra were obtained in an Ocean Optics spectrophotometer (Tampa, FL, USA), model USB 2000, equipped with an optical fiber, tungsten-halogen source, and silicon (350–720 nm) and germanium (720–1050 nm) detectors. The measurement of the Zeta Potential (ζ) was performed in the ZETASIZER Malver equipment (London, UK), model NANO ZS90, DTS1070 acrylic cuvette. For measurements, 1 mg of sample was dispersed in 1 mL of water (pH 7.0), and the suspension was kept in an ultrasonic bath for 480 s. Measurements were performed in triplicate, with 10 scans, and an equilibrium time of 120 s. Aliquots of water after treatment were studied by Inductively Coupled Plasma Optical Emission spectroscopy (ICP-OES, Perkin Elmer Optima 7300 DV, (Waltham, MA, USA)) for evaluating the content of Al and Ag against simple standard solutions. For X-ray photoelectron spectroscopy (XPS), a VersaProbe PHI 5000 spectrometer from Physical Electronics (Chanhassen, MN, USA), equipped with a monochromatic Al Kα X-ray source, was used. During the measurements, a dual-beam charge neutralization with an electron gun (1 eV) and an Ar ion gun (≤10 eV) were used for charge compensation of the sample surface.

### 2.6. Lake Water Collection and Treatment

Water collection was performed from the lake of the Cedeteg Campus/UNICENTRO (Guarapuava, Parana, Brazil), using 100 mL sealed and sterilized bottles provided by the Water Analysis Laboratory, UNICENTRO. All samples were collected at the same point, at 2 m from the lake margin. Collection, treatment, and physical–chemical and microbiological analysis were performed on the same day.

**Figure 3:**
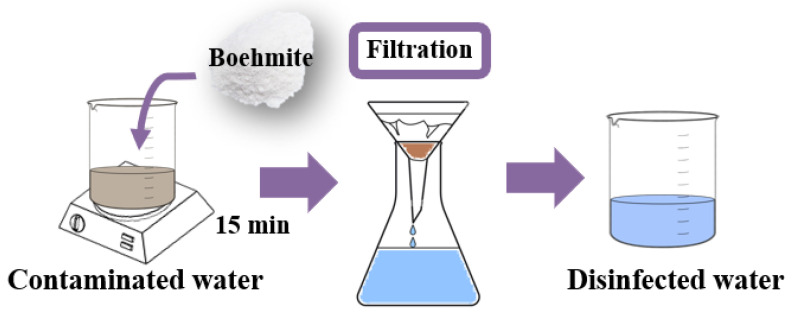
Schematic diagram used for lake water treatment.

The water treatment was carried out with the addition of 0.25 g of sample in100 mL of lake water. The boehmite and Ag-boehmite samples were tested separately to evaluate the performance before and after the surface decoration. The method used for disinfection was direct contact between the contaminated water and the samples for 15 min under constant agitation (500 rpm) at room temperature, followed by simple filtration, called the first aliquot (Figure 3).

The following aliquots (2nd, 3rd, 4th, and 5th) were carried out with the simple and direct filtration of lake water in the sample used in the 1st aliquot, to verify the possibility of reusing the powder samples. The treated water was collected in sealed and sterilized flasks for microbiological and physical–chemical analysis, according to the methodologies described in “Standard Methods for the Examination of Water and Wastewater” [36]. The evaluated parameters were total coliforms, pH, turbidity, and *Escherichia coli*, as shown in Table 1. The solid samples were oven-dried and characterized by Energy Dispersive X-ray Fluorescence (EDXRF) to analyze their composition after water treatment.

## 3. Results and Discussion

### 3.1. Scanning Electronic Microscopy with Energy Dispersive Spectroscopy (SEM/EDS)

**Figure 4:**
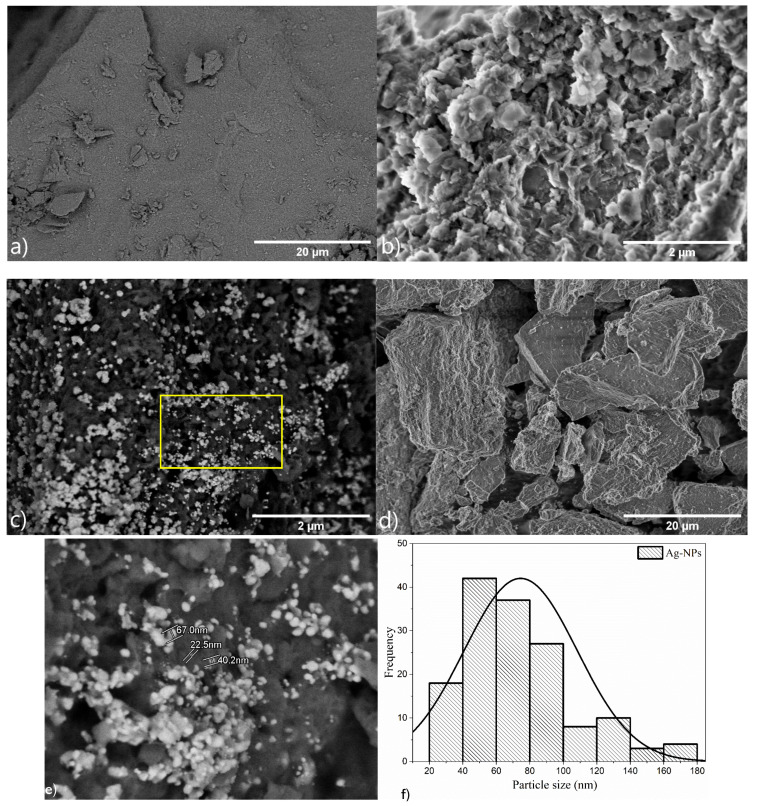
Scanning electron microscopy images of samples: (**a**) boehmite (LA detector), (**b**) boehmite (SE detector) (**c**) Ag-Boehmite (LA detector), (**d**) Ag-Boehmite (SE detector), (**e**) Ag-Boehmite (LA detector), the image is the magnification of the region inside the yellow rectangle in image (**c**), the bight nanostructures are Ag nanoparticles, and (**f**) histogram of Ag particle size distribution, the diameter of the Ag particles was measured on image (**e**).

**Figure 5:**
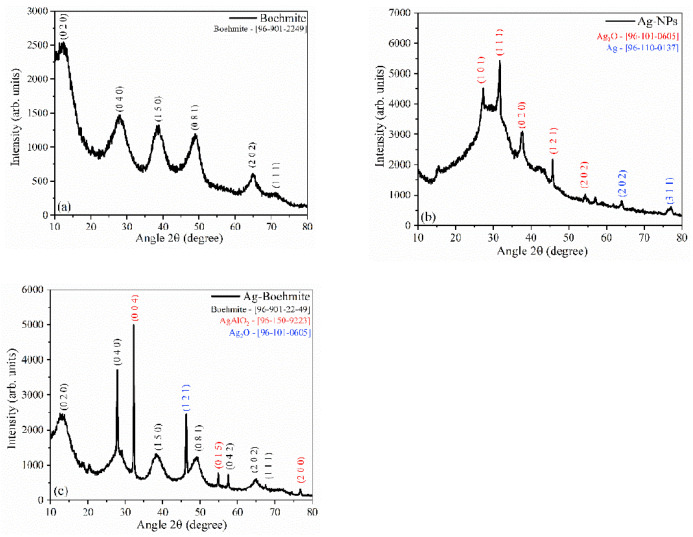
X-ray diffractograms of the samples: (**a**) boehmite; (**b**) Ag-NPs, and (**c**) Ag-Boehmite. The samples in powder form were placed directly in an appropriate sample holder for XRD analysis, scanning with an increment of 0.07°/s between 10° and 80° 2 theta.

The boehmite sample morphology (Figure 4a) presented irregular plates, as reported for products obtained using the same pH as this study [37]. To distinguish boehmite and Ag particles and to determine the Ag-NP density distribution, the Ag-loaded boehmite samples were visualized in the back scattered electron (BSE) mode of SEM (BSE-SEM) (Figure 4b,d). After the decoration with Ag-NPs (Figure 4c,d), the boehmite retained the same morphology; however, the Ag nanoparticles (bright contrast) were observed on the surface of the plates. The mean diameter of the silver particles was ~0.3 μm, corroborating the crystallinity data, and according to the histogram (Figure 5f), the mean particle size was about ~70 nm.

Table 2 shows the composition according to the energy dispersive spectroscopy, indicating the presence of aluminum, oxygen, and silver. Chlorine was a residue of the hydrochloric acid used for the digestion of the metallic can seal.

### 3.2. X-ray Diffractometry (XRD)

The X-ray diffraction (XRD) shown in Figure 5a confirmed the presence of the boehmite phase cubic spinel(γ-AlO(OH)), which was indexed according to the crystallographic chart [96-901-2249], using Match 3! software, and compared with the literature [38]. 

The XRD pattern of the synthesized silver nanoparticles is shown in Figure 5b; the presence of the Ag_2_O phase [96-101-0605] and metallic silver (Ag) [96-110-0137] were observed, indicating the reduction of silver ions. The XRD for boehmite decorated with Ag-NPs (Figure 5c) showed the presence of three crystallographic phases, with aluminum oxide-hydroxide as the majority phase [96-901-2249], the presence of the characteristic peaks of the AgAlO_2_ phase [96-150-9223], and Ag_2_O [96-101-0605].

The degree of crystallinity and particle size were calculated from the XRD patterns (Table 3). The boehmite had a degree of crystallinity of 60.4%, while the Ag-NPs had 19.6%. Decoration of boehmite with Ag-NPs promoted an increase in the crystalline Ag-Boehmite phase (80%), attributed to the presence of more crystalline oxides (AgAlO_2_ and Ag_2_O). The crystallite size was calculated for extreme form factors (k); the average size was 2.0–3.0 nm for boehmite, 21.0–31.0 nm for Ag-NPs, and the combination of boehmite with Ag-NPs resulted in an increase in crystallite size between 36 and 53 nm. The increase in crystallite size accompanied an increase in the degree of crystallinity, attributed to a strong structural interaction between the combined components, Ag-NPs, and boehmite.

### 3.3. Zeta Potential (ζ)

The boehmite (ζ = +1.58 mV) and Ag-boehmite (ζ = +1.67 mV) samples showed positive potential, confirming the tendency of aluminum oxide to be positively charged at a neutral pH [39]. On the other hand, the Ag-NPs had negative potential (ζ = −8.40 mV), thus suggesting that NPs were maintained by strong electrostatic interaction with the boehmite surface.

### 3.4. Water Treatment

The parameters presented in Table 3 were validated according to the Standard Methods for The Examination of Water and Wastewater—23th Edition—SMEWW [36]. According to this method, the limit of the microbiological parameters tolerated for total coliforms and *Escherichia coli* in the water is “<1 or absence”, and for the physicochemical parameters, the pH must be between 5.0 and 9.0, and the turbidity between null and 5.0.

The contaminated water was collected in a natural lake and presented a quantity superior to 2419.6 MPN of total coliforms. In addition, it showed the presence of 195.6 MPN of *Escherichia coli*. These microbes are strong indicators of the presence of feces in the water [40]. The boehmite sample was not able to eliminate the presence of total coliforms and *Escherichia coli* from the water; however, it reduced their presence by 77.4% (547.6 MPN) and 86.6% (26.2 MPN), respectively. The difficulty in eliminating E. coli lies in its cell wall characteristic, as the cell envelope contains several layers with unique compositions and structures, which combined form a multicomposite cell envelope structure resistant to environmental stresses [41]. However, the presence of Ag-NPs made the material capable of eliminating these bacteria, due to the antibacterial property of this metal [7]. Regarding the physical–chemical parameters, only the pH of the treated water samples was within the accepted limit.

**Figure 6:**
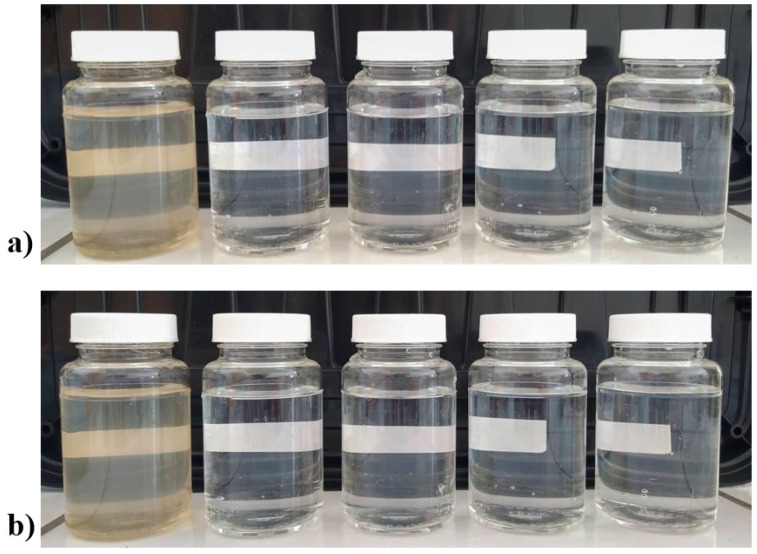
Digital photograph of flasks containing water treated with: (**a**) boehmite; and (**b**) Ag-Boehmite. First (left) flask not treated water, last flask water after 4th treatement.

Conversely, the Ag-Boehmite sample proved to be efficient in all parameters in the first aliquot (1st time), and in the reuses. it persisted within the limits only for *Escherichia coli* and pH, showing the possibility of using and reusing this material against this bacterium, since Ag^+^ ions are released to eliminate the pathogen, disrupting the cell walls of microorganisms, and inactivating the essential enzymes by damaging the DNA [42]. The boehmite sample proved to be very efficient in reducing the turbidity to acceptable levels (Table 4, Figure 6), and with the presence of Ag-NPs (Ag-Boehmite sample), it had permanent action against *E. coli*; however, it lost the ability to reduce total coliforms after the first use.

XPS was used to evaluate the chemical changes in the Ag-NPs sample after the water treatment. The results of the XPS analysis are shown in the supplement (Figure S1 and Table S1). After water treatment, there was an excess of carbon at the sample surface as expected. The FWHM of the Ag 3d core level slightly increased, which can be associated with a partial oxidation of the Ag nanoparticles.

### 3.5. Inductively Coupled Plasma–Optical Emission Spectrometry (ICP-OES)

The ICP-OES technique was used to monitor the amount of aluminum and silver in the water before and after disinfection (Table 5). The results indicated that there was no silver leaching into the water, with values below the detection limit, demonstrating that the synthesis of silver-decorated boehmite resulted in a stable material capable of disinfecting water without polluting [43], corroborating the zeta potential data.

### 3.6. Colorimetry (CIEL*a*b*)

The colorimetry of the samples was performed to obtain information about the color variation before and after the water treatment. In both samples the luminosity (L*) decreased, so the samples became less luminous after contact with contaminated water (Table 6). On the other hand, the samples showed a higher C* value, indicating that they became more saturated in color [44]. In comparison (∆E), for boehmite, there was a strong difference concerning its peer after water treatment and a very strong difference for Ag-boehmite [29].

**Figure 7:**
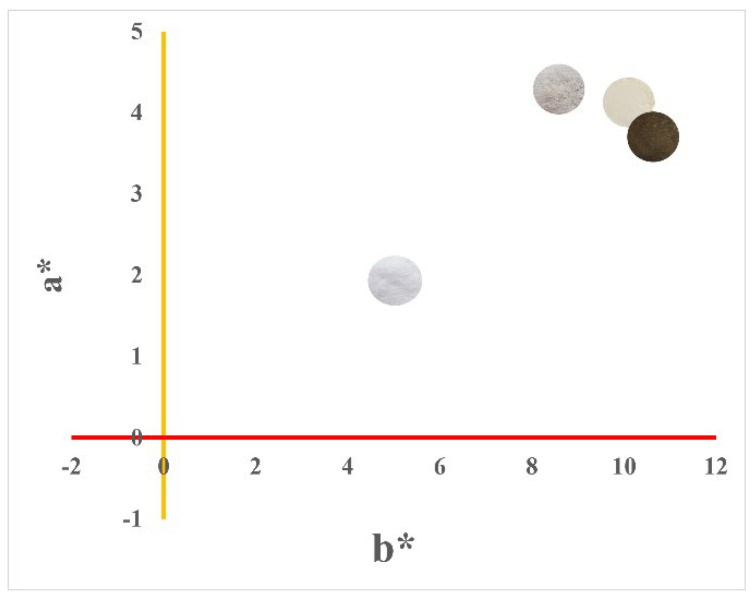
Graph of colorimetric parameters +a* (yellow) vs. +b* (red). Colorimetric measurements (L*a*b*) were performed on the respective samples in the form of powder.

All samples were positioned in the red/yellow color quadrant with positive a* and b* values (Figure 7). The trend towards yellow (a* more positive) observed in the materials is justified by the adsorption of the sulfur present in the water during the treatment [45].

## 4. Conclusions

The results show that the greener synthetic route used to obtain boehmite with a silver-modified surface was efficient. X-ray diffractometry (XRD) confirmed the phase (γ-AlO(OH)) and indicated that Ag deposition occurred on the boehmite surface, because there was no structural change observed, which was confirmed by scanning electron microscopy (SEM). The application as a disinfecting agent in the water was effective, as the treated water presented all the analyzed parameters within the established limits, emphasizing the absence of monitored microbes. In addition, there is the possibility of Ag-boehmite reuse, because even until the fifth reuse, it remained efficient against the gram-negative bacterium *Escherichia coli*.

## Figures and Tables

**Table 1:** Methods used in the analysis of the treated water and the main parameters evaluated.

Parameter Analyzed	Method
**Total coliforms**	SMEWW 9223 B- Enzymatic Substrate Coliform Test
***Escherichia coli***	SMEWW 9223 B
**pH**	SMEWW4500H + B—Electrometric Method
**Turbidity**	SMEWW 2130 B—Nephelometric Method

**Table 2:** Chemical composition of the samples according to EDS characterization.

Sample	% Weight				
	C	Al	O	Cl	Ag
Boehmite	2.8	37.4	53.5	6.3	-
Ag-Boehmite	1.5	57.1	33.2	4.6	3.6

**Table 3:** Crystallinity parameters (percentage and size) calculated from XRD.

Sample	Crystallinity (%)	Crystallite Size (nm)	
		k = 0.9	k = 1.3
Boehmite	60.4	2.1	3.0
Ag-NPs	17.9	21.2	30.7
Ag-Boehmite	80.0	36.3	52.4

**Table 4:** Microbiological and physicochemical analysis of the water before and after treatment.

Sample	Parameters			
	Total Coliforms (MPN/100 mL)	*Escherichia coli* (MPN/100 mL)	pH	Turbidity (NTU)
Lake Water (100 mL)	>2419.6	195.6	6.3	13.0
1st use Boehmite	547.5	26.2	5.1	0.7
reuse Boehmite	Presence	Presence	5.5	5.6
2nd reuse	Presence	Presence	5.8	5.7
3rd reuse	Presence	Presence	5.8	6.3
4th reuse	Presence	Presence	6.0	5.6
1st use Ag-Boehmite	<1	<1	6.0	4.8
reuse Ag-Boehmite	Presence	<1	6.1	5.8
2nd reuse	Presence	<1	6.1	5.9
3rd reuse	Presence	<1	6.1	5.2
4th reuse	Presence	<1	6.2	5.5

**Table 5:** Aluminum and silver concentration (ppm) in water after treatment.

Sample	Concentration (ppm)	
	Al	Ag
Contaminated water	0.371 ± 0.0028	−0.031 ± 0.0025
Boehmite	3.299 ± 0.0138	−0.025 ± 0.0019
Boehmite-Ag	0.082 ± 0.0012	−0.014 ± 0.0048

**Table 6:** Colorimetric parameters of samples before and after water treatment.

Sample	Colorimetric Parameters				
	L*	a*	b*	C	∆E
Boehmite 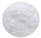	89.40	1.94	5.00	5.37	6.54
Boehmite (after) 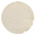	85.94	4.12	10.10	10.91	
Ag-boehmite 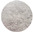	63.28	4.28	8.58	9.51	14.26
Ag-boehmite (after) 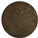	49.18	3.70	10.63	11.26	

## Data Availability

Not applicable.

## Supplementary Materials

The following supporting information can be downloaded at: https://www.mdpi.com/article/10.3390/nano12162771/s1; Table S1: Evaluation of surface chemical composition before and after (5th aliquot) water treatment; Figure S1: XPS 3d core level recorded on the Ag-NPs samples before and after water treatment.
