# Supplementary material for: Synthesis and Characterization of Boehmite Particles Obtained from Recycling: Water Disinfection Application
Source: Nanomaterials (Basel). 2022 Aug 12;12(16):2771. doi: 10.3390/nano12162771 (PMC9415003; doi:10.3390/nano12162771)
Supplement: Supplementary file 1 [file nanomaterials-12-02771-s001.zip › nanomaterials-1846281-supplementary.pdf]

nanomaterials

Supplementary Materials

Synthesis and Characterization of Boehmite Particles Obtained from Recycling: Water Disinfection Application

Dienifer F. L. Horsth [1,2], Julia de O. Primo [1,2], Nayara Balaba [1], Jamille S. Correa [1], Cristina M. Zanette [3], Douglas K. Silva [1], Carla Bittencourt [2,*] and Fauze J. Anaissi [1]

[1] Departamento de Química, Universidade Estadual do Centro-Oeste, Guarapuava 85040-167, Brazil
[2] Chimie des Interactions Plasma-Surface (ChIPS), Research Institute for Materials Science and Engineering, University of Mons, 7000 Mons, Belgium
[3] Departamento de Engenharia de Alimentos, Universidade Estadual do Centro-Oeste, Guarapuava 85040-080, Brazil
* Correspondence: carla.bittencourt@umons.ac.be

X-ray photoelectron spectroscopy:

Table S1. Evaluation of surface chemical composition before and after (5th aliquot) water treatment.

Sample	C at%	O at%	Al 2%	Ag 3d
Ag-NPs before water treatment	9.5	62.0	27.0	1.5
Ag-NPs after water treatment (5th aliquot)	22.0	54.0	23.5	0.5

The 3d Ag peak recorded on the sample after water treatment shows a second doublet (35% of the peak), this doublet component can be associated to the formation of Ag-O bonds during the successive water treatment steps.

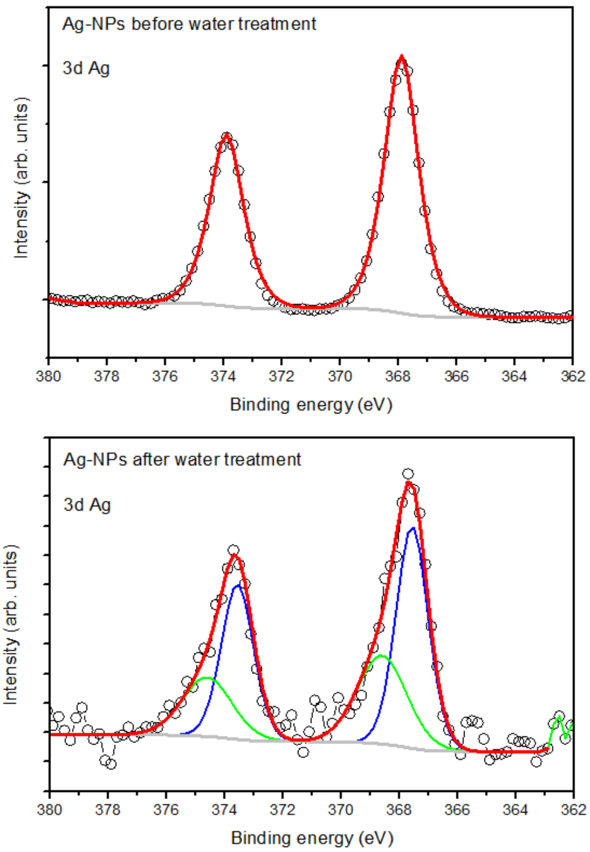

Figure S1. XPS 3d core level recorded on the Ag-NPs samples before and after water treatment. .
